# Augmented reality visualization of automated path planning for percutaneous interventions: a phantom study

**DOI:** 10.1007/s11548-022-02690-4

**Published:** 2022-06-23

**Authors:** Lovis Schwenderling, Florian Heinrich, Christian Hansen

**Affiliations:** Faculty of Computer Science and Research Campus STIMULATE, University of Magdeburg, Magdeburg, Germany

**Keywords:** Medical augmented reality, Automated path planning

## Abstract

**Purpose:**

Insertion point identification is a major challenge for percutaneous interventions. Planning in 2D slice image data is time-consuming and inefficient. Automated path planning can help to overcome these challenges. However, the setup of the intervention room is difficult to consider. In addition, transferring the insertion point to the skin is often prone to error. Therefore, a visualization for an automated path planning was implemented.

**Methods:**

A condition-based automated path planning was calculated with path length, distance to risk structures and insertion angle. The results were displayed on a phantom using projector-based augmented reality (AR) with an access point selection using the insertion needle. Two variants of the insertion visualization and three target displays were evaluated in a user study.

**Results:**

A visualization of insertion points with a representation of the path quality resulted in a choice of safer paths, compared with no insertion point display or no coding of the path quality. A representation of the target was preferred in the final survey, but did not perform better. A target display separate from the insertion point visualization reduced interferences between visualizations.

**Conclusion:**

A projector-based AR visualization of automated path planning results supports insertion point identification for percutaneous interventions. A display of the path quality enables the choice of safe access paths especially for unexperienced users. Further research is needed to identify clinical benefits and applicability.

## Introduction

Percutaneous interventions describe a group of procedures with minimized tissue injury. Compared to open surgeries, they result in smaller wound trauma, lesser pain and a faster recovery [1]. However, the altered visual and haptic feedback poses challenges. Since only a needle-like tool is inserted into the target, no endoscopic visual control can be performed. Access path planning prior to the intervention is therefore used to reduce the risk of complications. However, the expertise of the executing radiologist affects the outcome of the planning [2]. Planning in 2D slice image data is not intuitive [3] and assessing three-dimensional (3D) shapes is complicated [4]. Depending on quality requirements and case complexity, the process can be time consuming [5].

Approaches on automatic path planning were presented to allow fast and consistent identification of access paths [5]. A common approach is condition-based path planning, where access paths are determined based on hard and soft conditions. Hard conditions must be fulfilled to exclude unsafe paths. Soft conditions are used to determine the path quality and therefore allow for a rating [6]. High flexibility and adaptability result in a wide range of applications, e.g., drill paths in neurosurgery [7] or tumor ablations in the liver [4]. Drawbacks of automated path planning concern the adaptation to particular circumstances. Individual preferences, situation-specific conditions and previous experience cannot be taken into account [8]. Schumann et al. [9] therefore considered conditions for the positioning of equipment. However, this is only feasible for immovable objects. Furthermore, the insertion point has to be transferred to the skin after planning. This could cause inaccuracies in traditional planning as well as in automated path planning [10].

Automated path planning results are usually presented on a screen, where the final path decision is made by the radiologist. For example, insertion areas can be transparent [3] or colored [4, 11] on a model of the skin. Paths can also be represented by volumes [7, 12]. Augmented reality (AR) can be used to allow consideration of the intervention setup for the final path decision by the radiologist, as well as easier transfer of the path to the patient. AR describes the interactive and spatially registrated extension of reality. It can be used, for example, to display insertion points [13], anatomical models [14] or navigation aids [10] directly on the patient. Schwenderling et al. [15] showed automatic path planning results on a torso phantom using projector-based AR. The ability to choose a path from a selection of insertion points on the skin was preferred. However, missing information of anatomical context and target positioning was criticized.

Therefore, this work will investigate aspects for the visualization of the results of an automated path planning with AR. By displaying several insertion points on the skin, a path selection directly on the patient immediately before the start of the intervention should be made possible. While Schwenderling et al. [15] investigated the amount of displayed insertion points, there was no examination of how the paths should be displayed. Therefore, variations of an AR insertion visualization were evaluated for this work. To address the issue of missing anatomical context, this work will investigate an addition of a projected target visualization, combined with the slice image data on a screen. Underlying structures are often visualized as part of a navigation system. A common approach is using AR to display 3D models of target or risk structures [14, 16]. Path visualizations for navigated interventions can also indicate target positions [10]. A more abstract approach is the visualization of structures on an occluding surface. Outlines of target and risk structures and safety margins can be projected on organ surfaces [17], skin [13] or resection planes [18]. Different concepts for target visualization are considered in the context of this work and combined with the insertion visualization.

## Materials and methods

### Automated path planning

Because of the widespread use in the literature, a condition-based path planning was implemented. Only non-intervention specific conditions were used to allow for various application areas. For each aspect, one hard and one soft condition was considered (see Table 1). First, the phantom surface was scanned using rays starting from a predefined target in the liver. Information on the distance and angle as well as the collider hit was collected. A sampling rate of 9 points per square millimeter was determined experimentally based on accuracy and computing time. For each sample, representing a possible access path, hard conditions were evaluated first. If these were met, soft conditions were assessed. A condition quality value (CQV) between 0 and 1 was calculated for all soft conditions, with 1 representing optimal paths.

**Table 1:** Overview on the hard and soft conditions used for the automated path planning

	Hard condition	Soft condition
Distance to risk structures	No violation of risk structures	The more distance to risk structures the better
Path length	Paths must be shorter than the needle used	Shorter paths were rated better
Insertion angle	Angles with line-of-sight problems are excluded	Smaller angles to the imaging plane are rated better

#### Distance to risk structures

All risk structures were defined with a safety margin of 2 mm. If a path violated this space, the hard condition was not met and the path was excluded. The maximum evaluated distance was set to 1 cm, based on the maximum evaluated range in intraoperative distance visualizations [19]. To check the distance to risk structures, a cylinder was defined between the target and the insertion point. The radius was gradually increased up to the maximum evaluated distance. If an overlap occurred, the computation was stopped and the last radius was set as distance to risk structures.

$$\begin{aligned} CQV_{dist} \!=\! \frac{ distance~to~risk~structure - safety~margin }{ maximum~distance - safety~margin } \end{aligned}$$

#### Path length

The maximum path length was set to 15 cm, based on the needle used. The path length was evaluated relative to the vertical surface distance. This was determined by a ray cast from the target vertically upwards to the surface. A relative evaluation to a target-specific distance was used to not disadvantage deeper targets further away from the surface.

$$\begin{aligned}&CQV_{path}\\&\quad = 1 - \frac{path~length - vertical~surface~distance}{maximum~path~length - vertical~surface~distance} \end{aligned}$$

#### Insertion angle

The insertion angle denotes the angle between the evaluated path and the sagittal axis (vertical axis). Paths with an insertion angle $>60^{\circ }$ were excluded to prevent line-of-sight problems with optical tracking. For the soft condition, only the partial angle to the transverse plane was considered, as in-plane paths are easier to track. The conventional imaging plane is aligned with the transverse plane; however, it is possible to tilt imaging devices. Therefore, based on typical tilt angles of CT gantries, angles up to $25^{\circ }$ were rated as very good (linear mapping to the range [0.9, 1]). For larger insertion angles $\le 60^{\circ }$, a linear mapping was performed to the range [0, 0.9].

$$\begin{aligned}&CQV_{angle}\\&\quad = {\left\{ \begin{array}{ll} 1 - \frac{insertion~angle * (1 - 0.9)}{ tilt~angle} &{} \text {if }\,{insertion~angle} \le \, tilt \\ 0.9 - \frac{0.9 * (insertion~angle - tilt~angle)}{max~angle - tilt~angle} &{} \text {else } \end{array}\right. } \end{aligned}$$

All CQVs were clamped into the range [0, 1] and saved along with the texture coordinates of the insertion point. The path quality value for each insertion point was determined by assigning the minimum CQV for each point. The minimum was used instead of a weighted combination to better differentiate particularly good paths without trade-offs. The decision to take a risk due to benefits of another condition is left to the user.

$$\begin{aligned} {\text {path quality value}}=\min ({\text {CQV}}_{\mathrm{dist}}, {\text {CQV}}_{\mathrm{path}}, {\text {CPV}}_{\mathrm{angle}}) \end{aligned}$$

### Visualization

Two projected visualizations were combined to display possible insertion points and the target of the intervention. Additionally, a colored slice view showing the target structure and surrounding anatomy was always available. Only one slice for each anatomical plane, showing the center of the target, was displayed. Therefore, participants could not interact with the images. No medical image data or matching anatomy was available for the torso phantom used. Other models had to be utilized; therefore, no anatomical landmarks could be used. To allow transfer of the slice image data, reference points were added to the costal arches and the tip of the sternum. Yellow spheres were visible in the slice image data as well as in the projection. The color and structure of the reference points were chosen to minimize influence on the visualizations.

#### Insertion point visualization

**Fig. 1:**
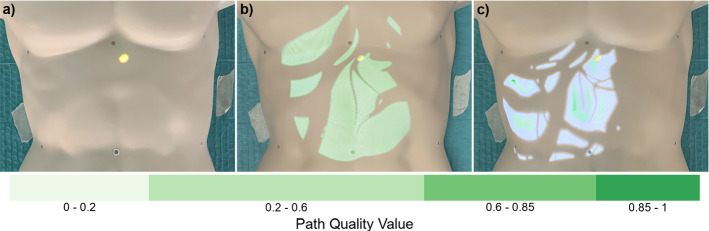
With the insertion visualization, possible insertion points that met the hard path planning conditions were projected onto the skin. For the *Area visualization* (**b**), no further information on path quality was given. In the *Full visualization* (**c**), insertion points were colored based on the associated path quality value, determined with soft path planning conditions. A green color scale, where darker shades represented better paths with higher path quality values, was used. For the baseline, no insertion points were displayed (**a**)

To visualize the path rating, all insertion points were divided into four categories. A classification based on the path quality value was made in order to enable a simple identification of path safety. Each class was represented by a color value (see Fig. 1). The color scale was determined by an online survey with 46 participants conducted prior to the study. Variations for three basic color scales (valid/ invalid paths: green/red, red/blue and blue/blue) were investigated. For each scale, versions with and without colored unsuitable paths as well as continuous and discrete types were evaluated. A discrete green-red scale, but without representation of unsuitable paths, was found to be best. Further improvements were made based on qualitative feedback. Thus, a green single hue color scale^1^, where darker shades encode better paths, was used (see Fig. 1).

For the insertion visualization, a render texture was generated with a vertex-and-fragment shader. First, the corresponding color of an insertion point was determined based on the path quality value. Then, the texture was colored at the texture coordinates of the insertion point. All other areas were rendered black and were therefore not visible in the projection. Interpolation between fragments was used to create a continuous surface. The finished texture was displayed on a 3D model of the skin. Depending on the target visualization, different shaders were used to render the black areas of the texture transparent or opaque. The visualization on the skin was then projected onto the phantom (see Fig. 1).

Two variants of the insertion visualization and a baseline, showing no insertion points, were evaluated in the study. The *Area visualization* showed possible insertion areas, without an indication of the path quality (see Fig. 1 b). For this purpose, the same color value was assigned for all possible paths when generating the texture, regardless of the path quality value. The *Full visualization* showed all suitable paths with a color-coded rating (see Fig. 1 c).

#### Target visualization

Three different concepts were chosen to represent the target structure, based on existing approaches. For each visualization, a representation of the target depth in the body was implemented.

**Fig. 2:**
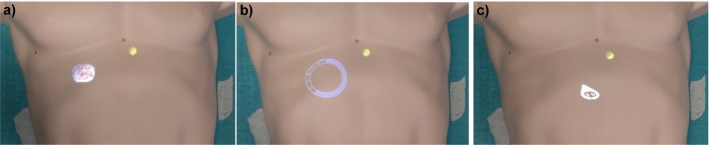
Overview of the three target visualizations used. **a** The *3D-Object* shows the target as a wireframe model with a reference line to the skin. **b** The *Ring* is projected onto the insertion visualization above the target. **c** The *Position Pin* is standing on the surface above the target, showing the depth in centimeters. Additionally, a slice view was shown on a monitor

In the concept *3D-Object* (see Fig. 2 a), the target was visualized with a spherical 3D model (diameter: 2 cm) at the target position. The size of the model was determined empirically in advance of the study to ensure visibility in the projection. A geometry shader with flat shading was used to display the model as a wireframe. The edges were colored white, the areas in between in red-brown. The colors as well as the wireframe representation were chosen to allow for a good differentiation from the insertion visualization. A vertical reference line was used to support the depth assessment [20]. Alternating black and white cylinders were displayed from the surface to the target. Each cylinder had a height of 1 cm and a diameter of 2.5 mm. The scaling was chosen as a compromise between visibility and low occlusion.

For the concept *Ring* (see Fig. 2 b), the position of the target was projected to the skin. With a ray from the target center vertically upwards, the position of the visualization and the distance to the skin was determined. There, a ring, consisting of 15 segments, was placed. A loading bar, filling the ring clockwise, was used to display the target depth. The segments served as centimeter markings. The visualization was implemented with two image files, one showing the outline and the other the loading bar. The loading bar was cut radially according to the target depth. To minimize occlusion, the images were displayed superimposed on the insertion visualization. For this purpose, the image of the ring was rendered into a texture. This was projected onto the insertion visualization with a virtual projector, placed above the target.

For the *Position Pin* concept (see Fig. 2 c), a position pin standing on the surface was used. Similar to the ring, the visualization was placed vertically above the target. The distance between target and skin was shown as a number in centimeters with one decimal place inside the pin. The visualization was implemented with an image file, which was always rotated toward the user to ensure good readability.

### Evaluation

**Fig. 3:**
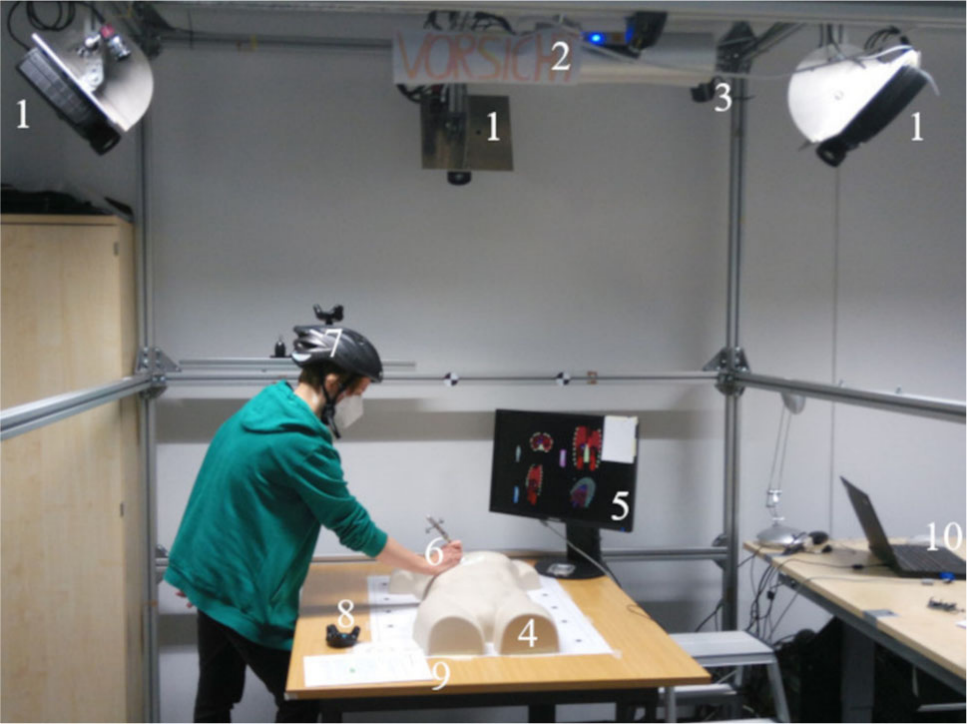
Overview of the technical setup. 1: Projectors, 2: Optical infrared tracking camera, 3: Base station for VIVE tracking (1/2), 4: Torso phantom, 5: Screen for slice image data, 6: Intervention needle with passive tracking geometry, 7: Helmet with VIVE tracker for head tracking, 8: VIVE tracker for registration, 9: Experimental instructions, 10: Work area of the experimental team

**Fig. 4:**
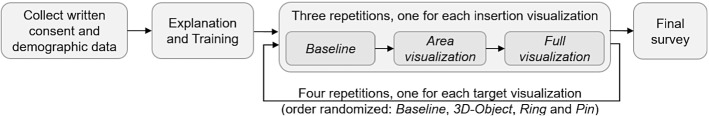
Workflow visualization of the study process

**Fig. 5:**
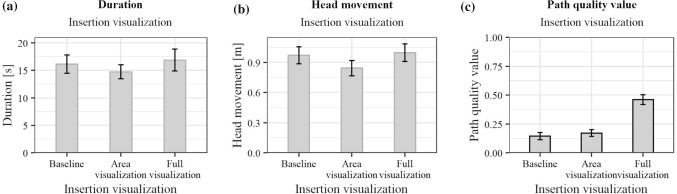
Effects of the three variants of the insertion visualization on the **a** *duration* , **b** *head movement*, **c** *path quality value*. Black outlines of the bars indicate significant results in the ANOVA

**Fig. 6:**
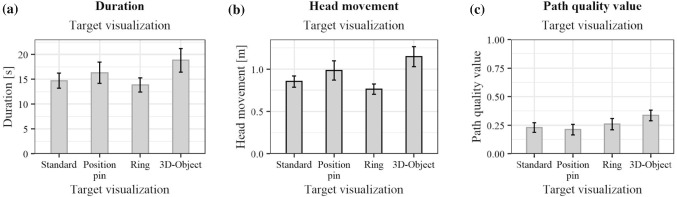
Effects of the four target visualizations on **a** *duration*, **b** *head movement*, **c** *path quality value*. Black outlines of the bars indicate significant results in the ANOVA

**Fig. 7:**
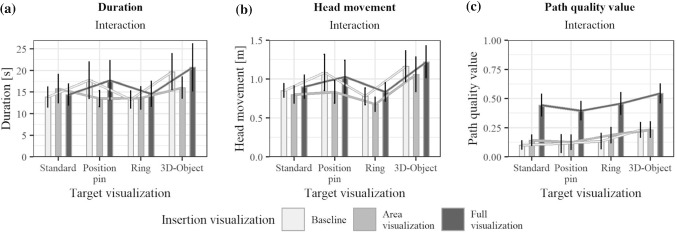
Interaction effects on the **a** *duration*, **b** *head movement*, **c** *path quality value*

A two-factor within-subject design study was conducted to investigate the insertion and target visualization. Three dependent variables were evaluated: the *duration*, the accumulated *head movement* and the *path quality value* of the selected insertion point. The *head movement* was measured to evaluate usage of the motion parallax depth cue. The *path quality value* was saved from the last intersection between surface and needle. It was used to evaluate the safety of the chosen paths. A higher *path quality value* was interpreted as a safer path. The visualizations were displayed on the surface of a torso phantom using a projector system. Three projectors (Barco F22WUXGA, Barco GmbH, Germany) were used to reduce occlusion and to increase the visibility of the projection (see Fig. 3 - 1). A surface scan and projector calibration was recorded using a photogrammetric measurement system (ProjectionTools, domeprojection.com GmbH, Germany). The generated model was used for projection mapping and creation of the visualizations. Projection mapping was done using the Unity Plugin based on Projection Tools (domeprojection.com GmbH, Germany). A head tracking to provide motion parallax depth cues was implemented using an HTC Vive Tracker (HTC Corporation, Taiwan) attached to a helmet (see Fig. 3 - 7). The position and rotation of the interaction tool was tracked with an optical infrared tracking camera (fusionTrack 500, Atracsys LLC, Switzerland, see Fig. 3 - 2). All tracking systems were registered to the projectors with registration markers in the origin of the projector coordinate system (see Fig. 3 - 8). 3D-models of organs, bones and blood vessels were selected from an anatomical database [21]. All methods were implemented using the game engine Unity (Unity Technologies, USA).

During the study, interpretation of slice image data was required. Therefore, participants had to be familiar with medical image data as well as general medical background information. As no knowledge of a specific intervention was required, medical students were invited. Thirteen medical students (7 female, 6 male) took part in the study. Participant age ranged from 23 years to 30 years (median: 25 years). Before start of the study, a training application was used to explain the visualizations and task. Participants were allowed to repeatedly perform the task and interactions. The participants were asked to select an insertion point on the skin using the slice images and the visualizations. They were instructed to critically analyze the displayed information. To select a point, an optically tracked interaction tool was placed on the torso phantom. The intersection point marked the selected path. It was shown on the surface in white to verify the chosen insertion point. If necessary, a reselection was possible until the selection was confirmed verbally by the participant, which ended the task. *Head movement* and *duration* were measured from the start of the task (signalized by the experimenter) to the signal of the participant. Participants were informed that the insertion point visualization was based on an automated path planning. They were repeatedly asked to critically question the information presented. For the target visualization, it was specified that the concepts did not represent path recommendations. The mismatch of anatomical landmarks of the torso phantom and the displayed anatomy was discussed. The participants were asked to use the reference objects instead. During the study, participants performed twelve tasks in total: one for each combination of target and insertion visualization. The process is displayed in Fig. 4. For each target visualization, the *Baseline visualization* was shown first, then the *Area visualization* and lastly the *Full visualization*. This order was intended to prevent participants from transferring additional information between the tasks. The order of the target visualizations was randomized between participants to avoid learning effects. Twelve comparable targets were defined and the corresponding insertion visualizations were calculated prior to the study. These were randomly assigned to the tasks. After all tasks had been completed, a final survey was conducted. On average, each participant took 45 minutes to complete the study. A two-factor analysis of variance (ANOVA) was conducted on the data to evaluate the main and interaction effects of the insertion and target visualization.

## Results

The results of the statistical analysis for the insertion visualization are presented in Fig. 5, and those for the target visualization in Figs. 6, and 7 shows the interaction effects of the independent variables. Table 2 shows the detailed results of the ANOVA.

No significant interaction effects were found. Therefore, the effect of the insertion visualization should not change depending on the target visualization and vice versa. An evaluation of the main effects is thus possible and is considered in more detail below. Disordinal interactions shown in Fig. 7 may be caused due to high data variance and would no longer occur with more participants. For *duration* and *head movement*, no statistically significant differences for the insertion visualization were found. Both variables show a similar distribution in Fig. 5a, b. This may be caused by the connection between *duration* and *head movement*: during a longer period of time, more *head movement* is possible. The *path quality value* showed statistically significant results for the insertion visualization. Safer paths were chosen for the *Full visualization*. The average *path quality value* is below 0.5. Best paths were, thus, not always chosen. This was also noticed during the study. Participants reported considering the slice visualization as well as anatomical landmarks. The insertion point visualization was felt to be helpful in validating a pathway decision. However, the decision was not made solely based on the insertion point visualization. The average *path quality value* chosen for the *Area visualization* is comparable to the *Baseline visualization*. Due to the lack of score representation, unsafer paths might have been chosen. The *Area visualization* was described as less helpful compared to the *Full visualization* because of a lack of comprehensibility. This suggests that the visualization of the path quality also contributes to a better understanding of the underlying planning algorithm. For the target visualization, differences have been found for the *head movement*. Participants moved most with the *3D-Object* and least with the *Ring*. This may be due to the increasingly used motion parallax depth cue for elements outside the projection surface. For the *3D-Object*, the reference line to the surface required additional time to count the sections to determine an exact depth. For the *path quality value*, no significant differences were shown. This suggests that the path decision was made based on the insertion point information and the slice view rather than the projected target visualization. However, during the final survey, it was frequently stated that a combination of insertion and target visualization was preferred. The *3D-Object* was most frequently selected as the most helpful concept.

**Table 2:** Summary of the ANOVA results ($\alpha < .05$) with degrees of freedom (df), *F*-value (F) and *p*-value (p) for significance. Effect size ($\eta ^{2}$) is given for significant results

Variable	Effect	df	F	p	$\eta ^{2}$
Duration	Main effect: *Target*	3	1.27	0.29	–
	Main effect: *Insertion*	2	0.41	0.66	–
	Interaction effect	6	0.26	0.95	–
Head movement	Main effect: Target	3	3.1	**0.03**	0.061
	Main effect: *Insertion*	2	0.99	0.38	–
	Interaction effect	6	0.07	1	–
Path quality value	Main effect: *Target*	3	1.82	0.15	–
	Main effect: *Insertion*	2	24.38	<**0.001**	0.253
	Interaction effect	6	0.06	1	–

Bold values indicate the significant results

## Discussion

In this work, the suitability of a projector-based AR display for insertion points was shown on a rigid phantom. However, further research is required to ensure clinical applicability. With a patient, soft tissue movements have to be considered. For example, an integration of respiratory compensation [22] is needed for most abdominal interventions to ensure high accuracy. Furthermore, setup and registration steps may interfere with the clinical workflow. The visibility of the visualization under surgical conditions must be ensured. During the intervention, the patient is covered in sterile covers. Thus, the insertion path planning has to be made before further preparations. Further investigation is therefore required with respect to the limitation of clinical workflow. While this work focused on the evaluation of visualizations, further research is also needed for the implemented path planning. Aspects of clinical suitability should be considered, e.g., correctness, performance and integrability into the clinical environment. Intervention-specific conditions should be integrated for further, more targeted studies.

The study was conducted with participants with little to no experience on percutaneous interventions. However, the planning outcome is influenced by the expertise of the radiologist. Inexperienced users are more reliant on assistance. This may have led participants to make decisions based primarily on the visualization rather than critically analyzing the insertion points as instructed. It was also described to be tempting to only ever choose paths marked as safest. However, when looking at the *path quality value*, good paths were not consistently chosen. Participants stated that they first determined paths using the slice image data which were then transferred to the phantom. The insertion visualization was used as a verification. Nevertheless, future research should be done with radiologists with varying expertise.

To avoid information transfer between tasks in direct succession, the order of insertion visualizations was not randomized. Participants might have been more familiar with the displayed information for *Full visualization* and thus performed better. However, the visualization order was not reflected in the results for *Duration* and *Head movement*. An effect is therefore considered unlikely. An inaccuracy may also have effected the time measurement, as subjects were asked to indicate completion of the task. This results in a variance in decision and reaction time that is added to the task completion time. However, since differences between concepts were investigated in a within-subject study design, an effect on the results is unlikely.

The mismatch between anatomical landmarks of the phantom and the anatomy shown was criticized. Furthermore, no palpation, e.g., of the ribs, was possible. Participants criticized that the slice view was not interactable and did not allow to scroll through the slices. The importance of the slice image data was emphasized by all participants to observe risk structures as well as for initial planning. A phantom with palpable anatomical landmarks and matching, interactable image data should therefore be used in further studies. In further studies, a projection of risk structures could be considered. Some participants used the insertion point visualization as a map of underlying structures. An integration of risk structures, comparable to risk maps [18], could also be considered.

## Conclusion

In this work, an AR visualization of the results of an automated path planning was investigated in a phantom study. Two insertion point visualizations combined with three target visualizations were evaluated and compared with baseline methods. It could be shown that a visualization of all insertion points with associated rating leads to participants choosing safer paths. The projection of a target visualization separate from the insertion point representation was preferred overall. Further research is needed to determine an optimal representation of the target. An additional display of the medical slice image data is indispensable. For the insertion point visualization, the advantages for inexperienced users were particularly emphasized. Further studies should also evaluate whether experienced radiologists can benefit from its use. The applicability of the results with a rigid phantom to patients needs to be investigated.

## Footnotes

1 C. A. Brewer, M. Harrower and The Pennsylvania State University. (2013). Colorbrewer: Color advice for maps, [Online]. Available: http://www.colorbrewer2.org (visited on 11/05/2021).
